# 731. CERTAIN-1: A Phase 3 Study of Cefepime-Taniborbactam Efficacy and Safety in the Treatment of Complicated Urinary Tract Infections (cUTI), including Acute Pyelonephritis (AP)

**DOI:** 10.1093/ofid/ofac492.022

**Published:** 2022-12-15

**Authors:** Paul C McGovern, Florian Wagenlehner, Leanne Gasink, Greg Moeck, Patrick L McLeroth, Mary Beth, Aaron Dane, Tim Henkel

**Affiliations:** Venatorx Pharmaceuticals, Malvern, PA; Justuf Liebeg University Diessen, Diessen, Hessen, Germany; LBG Consulting, Saint Davids, Pennsylvania; Venatorx Pharmaceuticals, Malvern, PA; Labcorp, Princeton, New Jersey; Venatorx, Malvern, Pennsylvania; Danestat Consulting Limited, Macclesfield, England, United Kingdom; Venatorx Pharmaceuticals, Malvern, PA

## Abstract

**Background:**

Carbapenem-resistant Enterobacterales and multidrug resistant *Pseudomonas aeruginosa* are global antimicrobial resistance threats. Cefepime-taniborbactam (FTB) is an investigational β-lactam/ β-lactamase inhibitor combination that is active against Enterobacterales and *Pseudomonas aeruginosa* expressing serine and metallo-β-lactamases. CERTAIN-1 (Cefepime Rescue with Taniborbactam in cUTI) evaluated FTB efficacy and safety in the treatment of cUTI.

**Methods:**

CERTAIN-1 was a randomized, double blind, double dummy, Phase 3 study comparing FTB to meropenem (MEM) in adults hospitalized with cUTI or AP. The primary endpoint was the composite (microbiologic and clinical) response at the test of cure (TOC) visit in the microITT population. Patients were randomized 2:1 to FTB 2.5g IV q8h or MEM 1g IV q8h for 7 days or up to 14 days in patients with bacteremia. The non-inferiority margin was -15.0% and a pre-specified test for superiority for the primary endpoint was performed following confirmation of non-inferiority.

**Results:**

A total of 661 patients were randomized and 436 patients (66.0%) were included in the microITT population, including 42.2% with AP and 57.8% with cUTI. Patients > 65 years represented 38.0% of the microITT population and bacteremia was present in 13.1% of patients. Composite success was achieved in 70.0% and 58.0% of FTB and MEM patients, respectively (Figure). FTB was statistically superior to MEM for the primary endpoint at the TOC (treatment difference [FTB-MEM], 11.9%; 95% CI, 2.4 to 21.6; p=0.0136) and statistical superiority was sustained at the late follow up visit (LFU). Analyses of secondary endpoints and subgroups were consistent with the primary efficacy analysis. Treatment-emergent adverse events (TEAEs) were observed in 35.5% of FTB patients and 29.0% of MEM patients. Serious adverse events occurred in 2.0% and 1.8% of FTB and MEM patients, respectively. The most common TEAEs were headache (FTB 6.1%, MEM 3.7%) and diarrhea (FTB 4.1%, MEM 2.3%).

**Conclusion:**

Following 7 days of therapy, FTB was statistically superior to MEM for the primary endpoint at the TOC. Composite success for FTB remained statistically superior to MEM at the LFU visit. FTB was safe and well-tolerated with a safety profile similar to MEM.

**Disclosures:**

**Paul C. McGovern, MD**, Paratek Pharmaceuticals: Stocks/Bonds|Venatorx Pharmaceuticals: employee|Venatorx Pharmaceuticals: Stocks/Bonds **Florian Wagenlehner, MD**, Glaxo Smith Kline: Advisor/Consultant|Spero Pharmaceuticals: Advisor/Consultant|Venatorx Pharmaceuticals: Advisor/Consultant **Leanne Gasink, MD, MSCE**, Amplyx Pharmaceuticals: Advisor/Consultant|CSL Behring: Advisor/Consultant|Pfizer: Advisor/Consultant|Spero Therapeutics: Advisor/Consultant|Venatorx Pharmaceuticals: Advisor/Consultant|Vera Therapeutics: Advisor/Consultant **Greg Moeck, PhD**, Venatorx Pharmaceuticals: Employee|Venatorx Pharmaceuticals: Stocks/Bonds **Patrick L. McLeroth, MD**, Labcorp: Full time employee|Labcorp: Stocks/Bonds **Mary Beth Dorr, PhD**, Merck: Past employee|Merck: Stocks/Bonds|Pfizer: Stocks/Bonds|Venatorx: Employee **Aaron Dane, MSc**, Amplyx: Advisor/Consultant|AN2 therapeutics: Advisor/Consultant|Artizan: Advisor/Consultant|Cidara: Advisor/Consultant|ContraFect: Advisor/Consultant|Correvio: Advisor/Consultant|Davolterra: Advisor/Consultant|Destiny Pharma: Advisor/Consultant|Entasis: Advisor/Consultant|F2G Limited: Advisor/Consultant|GSK: Advisor/Consultant|Humanigen: Advisor/Consultant|Kymab: Advisor/Consultant|Modis: Advisor/Consultant|Orca: Advisor/Consultant|Pfizer: Advisor/Consultant|Phico: Advisor/Consultant|Pled Pharma: Advisor/Consultant|Rare Thyroid: Advisor/Consultant|Roche: Advisor/Consultant|Scynexis: Advisor/Consultant|Sinovent: Advisor/Consultant|Spero Therapeutics: Advisor/Consultant|Transcrip: Advisor/Consultant|Venatorx: Advisor/Consultant **Tim Henkel, MD, PhD**, Venatorx Pharmaceuticals: Stocks/Bonds.

